# Ethno-medicinal uses and cultural importance of stingless bees and their hive products in several ethnic communities of Bhutan

**DOI:** 10.1186/s13002-023-00639-8

**Published:** 2024-04-10

**Authors:** Thubten Gyeltshen, Chet P. Bhatta, Tulsi Gurung, Pelden Dorji, Jigme Tenzin

**Affiliations:** 1 Department of Forest Science, College of Natural Resources, Royal University of Bhutan, Punakha, Bhutan; 2 Department of Biology, Radford University Carilion, 101 Elm Avenue, Roanoke, VA USA; 3 Department of Agriculture, College of Natural Resources, Royal University of Bhutan, Punakha, Bhutan; 4 Department of Animal Science, College of Natural Resources, Royal University of Bhutan, Punakha, Bhutan

**Keywords:** Meliponiculture, Relative cultural importance, Traditional knowledge

## Abstract

**Background:**

Indigenous and non-indigenous people in subtropical and temperate areas of Bhutan share an intricate relationship with stingless bees for diverse purposes including ethno-medicinal uses. Stingless bees hold significant importance in the realms of social, economic, cultural, and spiritual aspects. Bhutan's cultural traditions demonstrate a strong bond with the environment, exemplified by the regular use of honey from stingless bees for remedies such as treating the common cold, cough, and sore throat.

**Methods:**

Ethnographic research was conducted to document the ethno-medicinal uses and cultural importance of stingless bees in Bhutan. We deployed semi-structured interviews with stingless beekeepers and honey collectors including traditional healers who perform religious rituals for curing and preventing physical and mental illness.

**Results:**

We documented 22 different uses of stingless bee honey in food, medicine, veterinary medicine, crafts, beliefs, and religious purposes. The relative cultural importance (RCI) of stingless bees among Bhutan's ethnic communities was assessed through our calculations. It was determined that these bees hold notably greater significance for the *Lhotshampa* communities compared to other ethnic groups in Bhutan. This finding demonstrates the dependence of Hindu ethnic communities on natural resources in their everyday life. All participant communities largely exploit these bees through destructive extraction practices. They often find the natural nests in nearby forests, transfer them as a log hive to their backyards, and practice traditional meliponiculture.

**Conclusion:**

The ethnic communities of Bhutan use stingless bees for various purposes and the local knowledge are persistent. However, significant efforts should be made to address the ethno-medicinal, ecological, biological, and commercial perspectives of meliponiculture in Bhutan.

## Background

Bee forms a major part of pollinator communities with socio-cultural, economic, and ecological importance [1, 2]. Among social bees, the stingless bees (Hymenoptera: Apidae: Meliponini) are honey-making bees that are an integral part of many ethnic cultures, both past and present [3, 4]. They are deeply embedded in human culture and represent a natural source of food, crafts materials, medicine, and alternative sources of income [5–8], hence emphasizing a strong and robust relationship with humans across the course of history. Stingless bees primarily nest inside the forest cover [9], but several ethnic peoples around the globe practice a commercial stingless beekeeping technique called “meliponiculture” for supplemental income where they utilize or sell the hive products for food, ethno-medicinal, and cultural purposes [10, 11]. Meliponiculture is even well documented by pre-Columbian Maya civilization in Mesoamerica centuries ago in the form of ancient paintings [1, 12, 13].

There are about 500 described species of stingless bees from the tropical and sub-tropical regions of the world [7]. Stingless bees build a complex perennial colony, forage across varied landscapes, and are accountable for pollination of one-fourth of cultivated plants in the tropics and subtropics [14]. However, on-going habitat fragmentation [15] and other anthropogenic disturbances [16] negatively affect their diversity and abundance across the globe. It raises growing concerns about global food security, environmental sustainability, crop-based economies, and people’s livelihoods [17]. Although the Western Hemisphere of the New World, particularly Central America and South America, is the major hub for stingless bees [1, 2, 18], the Eastern Hemisphere also constitutes 20% of them in Indo-Mayan and Australian regions [19]. Unlike the well-studied apiculture industry in terms of *Apis* species in the Eastern hemisphere [20], taxonomy of stingless bees and the practices of meliponiculture are under-studied and poorly documented from several countries of the Indian sub-continent such as India, Nepal, Bangladesh, Sri Lanka, Pakistan, and Bhutan [21, 22]. Furthermore, most of the global bee literature is focused on honeybees and bumblebees resulting in a huge knowledge gap on the various aspects of stingless bees [23]. Stingless bees are only represented by 6% of scientific publication compared to other eusocial bees [24]. In this context, documenting the scientific information through ethnobiology and ethno-taxonomy is of crucial importance to further explore ecology, biology, and taxonomy of stingless bees at different regions of the world [25, 26].

Local traditional knowledge is a great resource for bee scientists and conservation biologists to uncover alternative conservation practices and to promote the status of the local pollinators [8, 27]. Stingless bees exhibit a great cultural importance to many ethnic and tribal populations around the world [23]. People keep stingless bees and use hive products such as honey, cerumen, propolis, and pollen for a wide variety of regions [8, 23]. In Nepal, the Tharu people, for instance, include bee broods in their diet alongside honey and pollen to enhance male fertility, while the *Chhetri* people serve stingless bees’ honey as a holy drink (locally called as “*panchamrit”*) during birth and death rituals [8]. Likewise, the *Yuqui* (a Tupi–Guaraní-speaking folk from the Chapare region of Bolivia) manufacture arrows for hunting by mixing of cerumen and plant resin [28] and many rural populations in Amazon and South America use the stingless hive products for self-consumption or as a supplemental income [23]. The ethnographic survey is considered as an important tool, enabling conservation biologists and melittologists to gather essential information about stingless bees. This is achieved through interviews with local informants, a process often overlooked in conservation policies [2, 23, 25]. While bee products are frequently obtained from wild colonies using destructive methods [29], cultural and ethno-biological data on stingless bees are accessible for various indigenous and non-indigenous populations across Asia, with a particular focus on Nepal and India [8, 30]. Information obtained from those ethnographic studies serves as an important starting point to promote sustainable meliponiculture. Beekeepers in the Karnataka region of India have achieved success in constructing various trial modern hives [29]. Scattered records show that local people keep stingless bees in several districts of Bhutan by bringing natural log hives from the forest to their backyard and use the hive products in their daily lives [33]. However, that information needs to be formally assessed, organized, and published to promote sustainable meliponiculture and conservation of stingless bees in Bhutan.

Literature records on stingless bees from Bhutan only mention certain aspects of systematics and distributional records, providing scant details on their uses, cultural values, and other biological information. Two species of stingless bees, *Tetragonula gressitti* (Sakagami) and *Lepidotrigona arcifera* (Cockerell), were recently reported from Bhutan [33]. Local communities identify them as “*Kalo Putka*” (black color stingless bee) and “*Poilo Putka*” (yellow color stingless bee), respectively, and keep them in traditional hives and use them in multiple forms of traditional medicines.

Integrating traditional knowledge in biological research can enrich the natural history of the species by adding a plethora of cultural information gathered from the people who have been living in proximity for centuries [31]. In addition, they might provide us with additional ecological and biological information. However, in Bhutan, much work has been done to document the traditional uses of medicinal plants and plant products [31, 32], but there is a definite lack of ethno-entomological works related to stingless bees. Moreover, rural–urban migration and immigration trends have been comparatively high in recent years and the young generation undervalues the traditional knowledge transmitted orally from their elders [34]. To conserve traditional knowledge and promote the sustainable conservation of the extant species of stingless bees in Bhutan, it is crucial to document such traditional knowledge regarding ethno-medicinal and cultural uses. The objective of this study is to analyze the semi-structured interviews from several ethnic communities in Bhutan to better understand the cultural and traditional ethno-medicinal knowledge of stingless bees and meliponicultural practices performed by them. We also discussed the future direction of meliponiculture for the sustainable use of these stingless bees in Bhutan and how this traditional knowledge might transform and evolve into formal commercial meliponiculture. Additionally, we also conducted quantitative assessment to define the relative cultural importance of stingless bees for each ethnic community and their role for conservation of stingless bees in Bhutan. Therefore, we present, for the first time, the ethno-medicinal applications, cultural significance, and domestication practices of various stingless bee species within diverse ethnic communities in Bhutan. This research paper aims to supplement the national inventory of bee resources and traditional knowledge, contributing to their sustainable management in Bhutan.

## Methods

### Study area

**Fig. 1:**
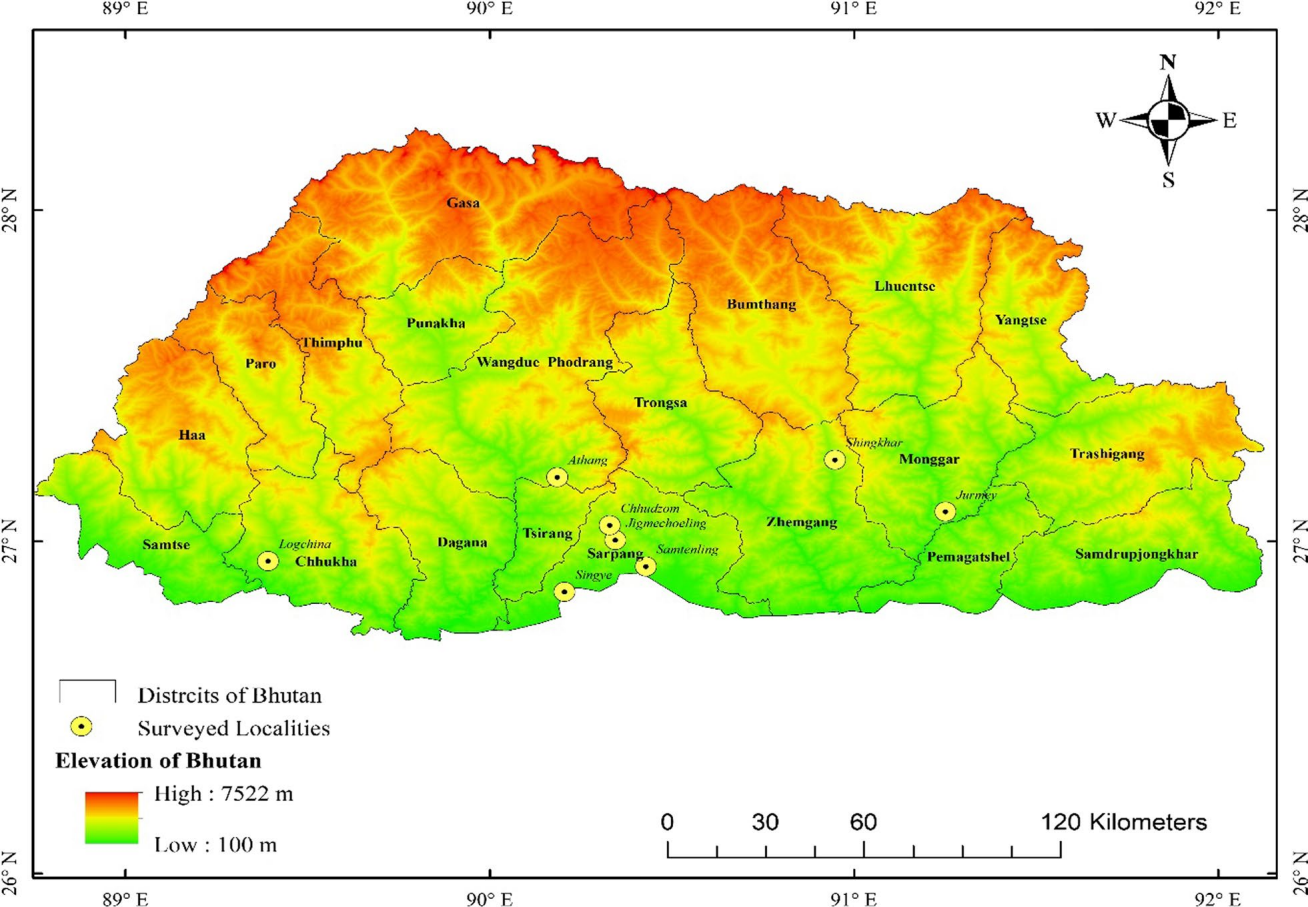
Map of Bhutan showing the study area, *Gewogs* (blocks) under Chhukha, Wangdue Phodrang, Sarpang, Zhemgang, and Mongar *Dzongkhags* (districts)

This study was conducted in five *Dzongkhags* (districts) and eight *Gewogs* (blocks) in Bhutan: Chhukha (Logchina) from western region, Sarpang (Chhundzom, Jigmecholing, Samteling, and Singye), and Zhemgang (Shingkhar) from south central, Mongar (Jurmey) from eastern region, and Wangdue Phodrang (Athang) from west central region as shown in Fig. 1 and Table 1.

**Table 1:** Geographical information of the study area

Region	District	Block	Coordinates		Elevation (m) at which the stingless bee hives were found
			Latitude	Longitude	
Western	Chhukha	Logchina	89.390660	26.940370	1060
West-central	Wangdue Phodrang	Athang	90.184973	27.192946	1545
South-central	Sarpang	Chhundzom	90.329298	27.047724	1848
		Jigmecholing	90.344170	27.003140	967
		Samtenling	90.427960	26.924060	638
		Singye	90.204752	26.847440	301
	Zhemgang	Shingkhar	90.947240	27.245140	1176
Eastern	Mongar	Jurmey	91.250710	27.089006	1188

### Ethno-zoological information collection

**Fig. 2:**
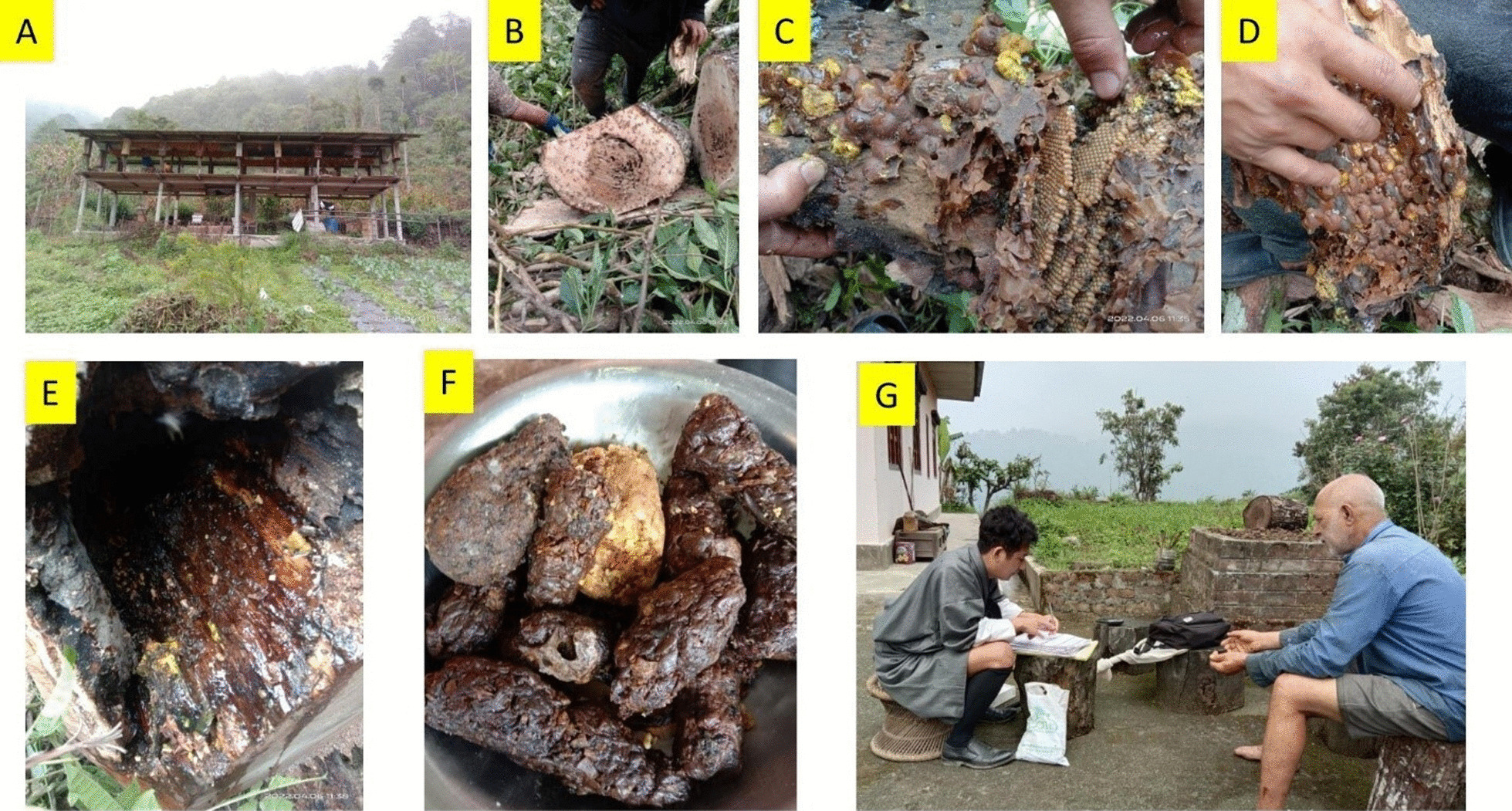
**A** Domestication of stingless bee in Chhudzom Gewog. **B**–**E**. Destructive extraction of stingless bees' hive products from wild colonies. *F* Wax and propolis collected for traditional use. **G** Interviewing with the Bhawan communities of Bhutan in Chhudzom Gewog (Senior author, on the right)

Bhutan is multi-ethnic and multi-lingual kingdom [35]. The primary ethnic groups in Bhutan include the *Ngalops*, of Tibetan descent, and the *Sharchops*, who are descendants of indigenous Tibetan people closely linked to the *Monpas* [36]. Another distinctive ethnic group in southern Bhutan is the *Lhotshampas*, descended from Nepalese origins (Fig. 2), and they inhabit the lowlands. This ethnic group is further divided into five sub-ethnic categories: the *Bhawan*, the *Gurung*, the *Tamang*, the *Monger*, the *Rai*. Each of them speaks their own dialects and has their own unique culture [36]. Likewise, the *Khengpas* are another distinct group from the south-central region of the country [37, 38].

The field survey for this study was conducted during the spring and summer months of 2022. Semi-structured interviews were conducted with 55 informants from five districts and eight blocks described in the study area. We followed all procedures and the code of ethics published in International Society of Ethnobiology Code of Ethics [39]. The criteria for selection of the informants were that they should have knowledge of the stingless bees in terms of types, uses, the location or ecology of the stingless bees in the wild, nesting behavior and the management practices. Therefore, the network of informants was built by the recommendations of *Gups* (Head of the block*)*, *Mangmis* (*Gup* assistant), and the informants themselves by using the snowball-sampling method [40, 41]. They belong to eight ethnic communities consisting of the *Bhawan*, the *Gurung*, the *Tamang*, the *Monger*, the *Rai* belonging to the larger category of the *Lhotshampas* and the *Ngalop*, the Sharchop, and the *Khengpa* (Table 2). The informants vary among the blocks, districts, and ethnicities indicating the prevalence of ethno-biological knowledge among different ethnicities. Informants consist of 50 males (90.9%) and 5 females (9.1%). Their age ranges from 21 to 74 years, and the majority (49%) of the informants were of the age 51–65 years. All those informants who could just read and write were considered literate (2.7%) and 67.3% were illiterate. The majority (70.9%) of the informants practice Hinduism followed by Buddhism and Christianity (Table 3). All the informants (100%) are farmers, mostly practicing integrated farming constituting crop and livestock production. Our informants either domesticated stingless bees in their backyard or collected stingless bee honey and honey products from wild colonies from their natural habitat. Interviews were recorded by using a Sony ICD-UX570 voice recorder in native languages, were later translated, and transcribed for the data analysis. Data analysis was done using computer-assisted software like Microsoft Excel and SPSS.

**Table 2:** Ethnicity of the informants

District	Block	Ethnic community	No. of informant from each block
Chhukha	Logchina	*Rai*	16
Sarpang	Chhudzom	*Rai*	7
		*Gurung*	5
	Samtenling	*Rai*	3
		*Bhawan*	3
	Jigmecholing	*Bhawan*	5
		*Tamang*	3
		*Monger*	1
	Singye	*Monger*	2
		*Tamang*	2
Mongar	Jurmey	*Sharchop*	2
Zhemgang	Shingkhar	*Khengpa*	2
Wangdue Phodrang	Athang	*Ngalop*	4
	Total		55

**Table 3:** Demographic profile of the informants

Characteristics	Categories	Informants (%) n = 55
Gender	Male	90.9
	Female	09.1
Age range	21–35	14.0
	36–50	35.0
	51–65	49.0
	> 65	02.0
Education	Literate	32.7
	Illiterate	67.3
Religion	Hindus	70.9
	Buddhist	20.0
	Christian	09.1
Profession	Farmers	100.0

The first author (TG) who is a native of Bhutan adopted a participant observation method [42], where he facilitated the interviews with informal conversations. The first author has good command over the national language of Bhutan, Dzongkha which is used to interview Dzongkha speaking *Ngalops* of Wangdue Phodrang. Lhotshampa (Nepali language) was used in all the blocks of Sarpang and Logchina of Chhukha. Sharchopkha dialect was used in the Mongar district. The fourth author was involved in an interview in Shingkhar, Zhemgang in local dialect Khengkha. Each interview lasted for a maximum of 30 min.

The National Biodiversity Centre, the national focal agency for inventory and documentation of traditional knowledge associated with biological resources reviewed this study and provided approval both for field research and publication.

### Ecological and socio-economic features of the study site

Logchina block in the Chhukha district has an area of 70.4 sq. km., 12 small villages, 430 households, and a population of 2500. The *Ngalops* and the *Lhotshampas* are the two major ethnic groups inhabited in this block. The livelihood in this block is supported by cash crops such as cardamom, ginger, areca nut, mandarin, and organic vegetables [43]. The elevation of this block ranges from 600 to 1200 m. above sea level and has subtropical climatic conditions [44]. Stingless bees are domesticated as one of the sources of income by some of the *Lhotshampas* farmers in this block.

Athang is one of the remotest blocks in the Wangdue Phodrang district. It lies at an elevation ranging from 700 to 3500 m. above sea level and characterized by dry subtropical climate. It has an area of 746 sq. km., 198 households, and a population of 793. The Ngalops constitute the major ethnic community residing in this block. This place is best known for its smoked fish (locally known as *Nga dosem*). Paddy, maize, wheat, barley, buckwheat, potato, and organic vegetables are the major cash crops in the area to support their livelihoods [45]. Jurmey block in the Mongar district is characterized by the subtropical climate. It has an area of 55 sq.km., 304 households, and a population of 2646. The *Sharchops* constitute the major ethnic community residing in this block. Maize is the main crop in this block, and it faces acute shortage of water as most of the settlements are on hilltops and the water sources are in the valley [46]. Shingkhar block is located at a higher altitude in a remote part of the Zhemgang district and well characterized by cooler climatic conditions. It has an area of 309 sq.km., 244 households, and a population of 960. The *Khengpas* people are the major inhabitants in this block and mostly dependent on livestock for their livelihood. This block is very rich in biodiversity and cultural heritage with numerous traditional and religious festivals [47]. The *Ngalop*s, the *Sharchops,* and the *Khengpas* people follow Buddhism as their religion. They practice extracting stingless bees’ hive products from natural wild nests and use them for medicinal purposes, yet they do not undertake domestication.

Chhudzom, Jigmecholing, Singye, and Samtenling blocks in the Sarpang district are inhabited by the *Lhotshampas* including the *Bhawan*, the *Gurung*, the *Tamang*, the *Mongar*, the *Rai* people. The *Lhotshampas* ethnic communities follow Hinduism as their religion. Chhudzom has a humid and wet subtropical climate. It covers an area of 222 sq. km., 637 households, and a population of 4422. Jigmecholing has an area of 501 sq.km., 698 households, and a population of 5716. It lies at an altitude of 1200–1500 m. above sea level. Singye has an area of 232 sq. km., 287 households, and a population of 1824. It lies at an elevation of 249–900 m. above sea level. Similarly, Samtenling has an area of 55 sq.km, 467 households, and a population of 3068. It lies at an elevation of 190–380 m. above sea level [48]. Agriculture is a significant economic sector in the Sarpang district to support the livelihoods of the *Lhotshampas* people. The *Lhotshampas* community in the above-mentioned blocks collects the natural nests of stingless bees, brings them to their homes in the form of log hives, and domesticates them in their backyards. They also practice destructive harvesting hive products from natural nests and utilize them in many ways.

### Quantitative evaluation of ethno-zoological data

We analyzed the ethno-medicinal uses and cultural importance of stingless bees and their hive products used by the different ethnic communities in Bhutan by grouping all the mentioned uses into several categories (#C) such as food, medicine, veterinary medicine, crafts, beliefs, and religious uses. Within those categories, specific uses (#U) of stingless bees and their hive products are sub-grouped (Table 5).

A “researcher tally” method as described by Philip [49] was employed to group specific uses of stingless bees and their hive products into several categories. In calculating the relative cultural importance (RCI) index, we adhered to the methodology outlined by Gonzalez et al. [50], employing a modified index derived from Bennett & Prance [51], which is commonly used to assess the RCI of medicinal plants. The index is calculated based on the sum of the proportion of the number of use categories (C) and the proportion of the number of specific uses (U) multiplied by 50 ([C + U] × 50). The output of the index is expressed on a scale from 0 to 100. Kruskal–Wallis test and Dunn’s multiple comparison tests were used to assess the relative differences in the RCI among different ethnic communities [8]. Additionally, we employed the mention index to evaluate different categories of uses, determining the proportion of mentions for a specific category divided by the total number of interviews [52].

### Collection and preservation of sample

Fifteen worker bee specimens were collected from each nest we encountered both in the forest and under domestication. Bee specimens were collected by using a sweep net [53]. The collected bee specimens were kept in vials containing ethanol in the field. Bee specimens were picked up by pointed forceps and pinned in the meso-scutum on the thorax with the help of the pinning block in the laboratory setting. After pinning, each bee specimen was dried and kept inside the specimen box for further morphometric measurement and identification process. Photographs and morphometric measurements were taken in the National Biodiversity Centre laboratory in Serbithang, Bhutan. After the identification of bee specimens, the identification label, the locality label, and the collector label were provided to each specimen.

### Morphometric measurements

Key features for identification are based on Sakagami [54] as cited in Rasmussen [21]. All morphological measurements were recorded in micrometer (mm). The measurements were taken with an image acquisition-programmed zoom-stereo microscope. Terminology and measurements follow as explained in Michener [7] and Ruttner [55]. The indices and their abbreviations used are: (1) total body length (BL), (2) head length (HL), (3) head width (HW), (4) length of clypeus (LC), (5) length of scape (LS), (6) length of mandible (LM), (7) length of forewing (LF), (8) width of forewing (WF), (9) length of hind wing (LH), (10) width of hind wing (WH), (11) width of tibia (WT), (12) length of basitarsus (LB), (13) length of meso-scutum (LM), (14)length of tibia (LT), and (15) width of basitarsus (WB). The observations pertaining to the bilateral body parts such as eyes, antennae, legs, and wings were taken on the right side of the body part. Using forceps, a bee sample from specimen box was picked and was placed under stereomicroscope for measurement of total body length and width. After recording, the sample was mounted under image acquisition-programmed zoom-stereo microscope for further morphometric measurement. After morphological characterization, the photographs were taken for further identification and documentation.

## Results

### Common names of stingless bee in Bhutan

The ethnic communities in Bhutan recognize stingless bees with varying common names (Table 4). The people of western Bhutan (the *Ngalops*) called them *Chuepjaam* or *Tabjaam*, while the people of eastern Bhutan (the *Sharchops*) called them *Ringbu* (‘*Ring*’ means the entrance tube*, ‘Bu’* means insect*;* thus*, Ringbu* means insect with entrance tube). The people of Zhemgang (the *Khengpas*) recognize them as *Chulingma*. Stingless bee in southern Bhutan is popularly identified as *Putka* in general, but different communities have specific local names: *Lepiditrigona arcifera* is known as *Poilo putka* (Yellow stingless bees) in Chhudzom, while it is called *Sheto putka* (White stingless bess) in Jigmecholing, Logchina, and Singye. On the other hand, *Tetragonula gressiti* is identified as *Kalo putka* (Black stingless bees). Both species are in use for medicine and other purposes without any distinction; however, the medicinal properties of *Kalo putka* are claimed to have more medicinal properties. The morphometric measurements and nest architecture confirmed the species being the *Tetragonula gressiti and Lepiditrigona acifera.*

**Table 4:** Local names of the stingless bees in different ethnic communities

District	Block	Ethnic communities	Common names	Scientific name
Wangdue Phodrang	Athang	*Ngalops*	*Chuepjaam or Tabjaam*	*Lepiditrigona arcifera*
Chhukha	Logchina	*Lhotshampas*	*Sheto putka* (white putka)	*Lepiditrigona arcifera*
Zhemgang	Shingkhar	*Khengpas*	*Chulingma*	*Lepiditrigona arcifera*
Sarpang	Chhudzom	*Lhotshampas*	*Poilo putka* (yellow putka)	*Lepiditrigona arcifera*
Sarpang	Jigmechoeling	*Lhotshampas*	*Sheto putka* (white putka)	*Lepiditrigona arcifera*
Sarpang	Jigmechoeling	*Lhotshampas*	*Kalo putka* (black putka)	*Tetragonula gressiti*
Mongar	Jurmey	*Sharchops*	*Ringbu*	*Lepiditrigona arcifera*

### Ethno-medicinal uses

We recorded 22 specific uses of stingless bees across all the ethnic communities and grouped them into six categories of use adopted in this study (Table 5). Both species are used for medicine and other purposes throughout the country. In general, *Kalo Putka* has a wider habitat range in the country and visits significantly more flower species to collect pollen and nectar compared to white species [33]. The local people believe that the honey collected by the black species has a higher medicinal value compared to the white species. However, both species find extensive applications in medicine and various other uses throughout the country. The *Bhawan* and *Rai* communities incorporate both pollen and honey into their diet, whereas *Gurung* and *Tamang* individuals exclusively use honey. The majority of informants noted oral consumption of stingless bee honey or its utilization as a key ingredient in traditional medicine. A quarter of the informants reported enhancing the therapeutic properties by adding ingredients like lemon juice, ginger, and Apis honey to address various ailments such as the common cold, tonsillitis, gastritis, common coughs, typhoid, allergies. Notably, the ethnic communities in southern Bhutan employ honey as a massage oil to alleviate pain associated with injuries and backaches among the elderly. Informants also utilize honey for the treatment of burns, fresh cuts, wounds, conjunctivitis, allergies, ringworms, and chickenpox (Table 5). The informants from southern Bhutan specifically mentioned that *“they would burn a small portion of the entrance tube and inhale the smoke to treat dizziness and nausea.”*

**Table 5:** Categories of use (#C), specific uses (#U), and mentioned indexed for each use #M) documented for the stingless bee in eight ethnic communities in Bhutan

Categories of uses (#C)	Specific uses (#U)	Mentioned index(#M)
Food	Honey (H)	6
	Pollen (P)	4
Medicine	Common cough and cold (H)	31
	Stomach ulcer (H)	24
	Pain due to injuries (H)	23
	Conjunctivitis-applied (H)	22
	Burns in skin or cut (H	23
	Ringworms (H)	4
	Tonsil (H)	7
	Chickenpox (H)	4
	Nausea/vomiting (E)	10
	Pneumonia (H)	9
	Gastritis (H)	12
Veterinary medicine	Food and mouth disease (H + P)	7
	Necrosis (H)	4
Crafts	Resin as a sealing glue in pots (R)	6
	Wax for manufacture candles (W)	5
Beliefs	Paste made up of entrance tube to protect from snake bite (E)	4
	Amulets made up of entrance-tube paste to protect from the evil spirit (E)	15
Religious purpose	*“Puran”* ritual (H)	5
	Ritual related to offering local deities (H)	3
	*“Satyanarayana puja”* (H)	2

For each medicinal use, the type of bee product used is provided in parentheses. *E* Entrance tube, *H* Honey; *P* Pollen; *W* Wax; *R* Resins

**Fig. 3:**
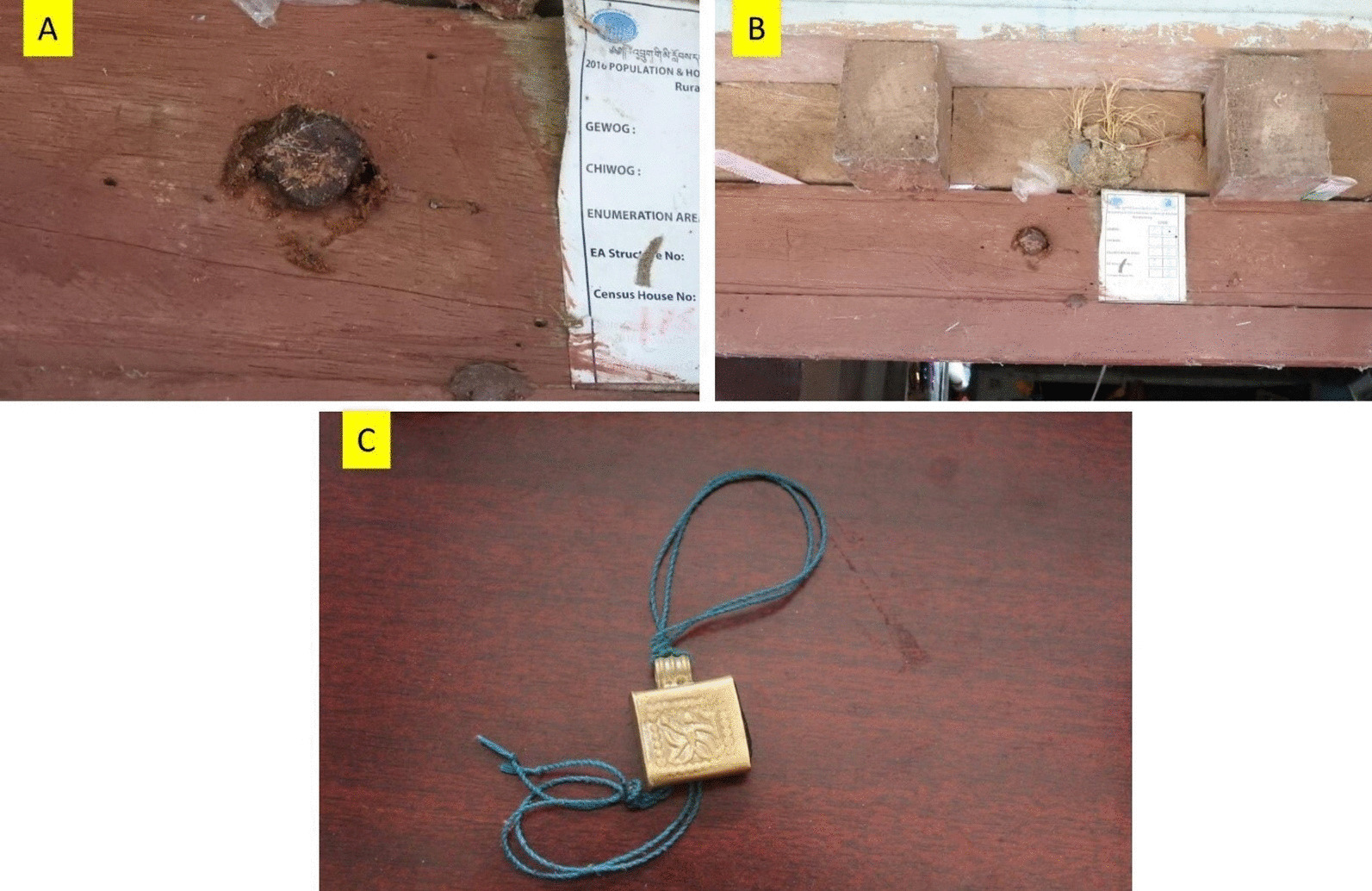
**A** and **B** Portion of entrance tube crushed and pasted above the door by the *Rai* communities in Logchina Gewog, Bhutan. **C** Local people make an amulet which consist of a small portion of the entrance tube and wear them around the neck

The *Bhawan* and the *Rai* people use honey to treat cattle diseases such as foot and mouth disease (FMD) and toe tip necrosis. They feed the raw honey mixed with pollen to treat FMD, while they feed and apply raw honey to the infected area to treat toe tip necrosis. Additionally, the *Bhawan* and the *Rai* people from Sarpang and Chhukha regions use wax and resins as well. They use resin (propolis) as a sealing glue, and wax is used to manufacture candles. One of the informants from Sarpang reported that *“the candles manufactured from the wax mixed with resins can last for 3 to 4 days.” Rai* and *Bhawan* people have a tradition of pasting a small portion of the entrance tube around the machete before they leave their house for their daily farming work. It is believed to protect them from snake bites, while they are working in agricultural fields or in the forests. The *Rai* communities practiced pasting a small portion of the entrance tube on the door (Figs. 3A and B) and making an amulet (called as “*Buti”*) (Fig. 3C) which they believed would protect them from the evil spirit. It illustrates the diverse and intricate historical relationship between people and the stingless bee in different ethnic communities of Bhutan.

The several ethnic communities of Bhutan use the honey of stingless bees in their religious activities on several occasions. The Rai community incorporates honey from stingless bees in the "Naya dhawta" ritual, which is associated with offerings to newly revered local deities. On the other hand, the *Bhawan* people use honey in “*Satyanarayana puja,”* and “*Puran”* (religious rituals for the recitation of the Hindu legends) to worship Lord “*Vishnu”* to fulfill their wishes and desires.

#### Mention index

**Fig. 4:**
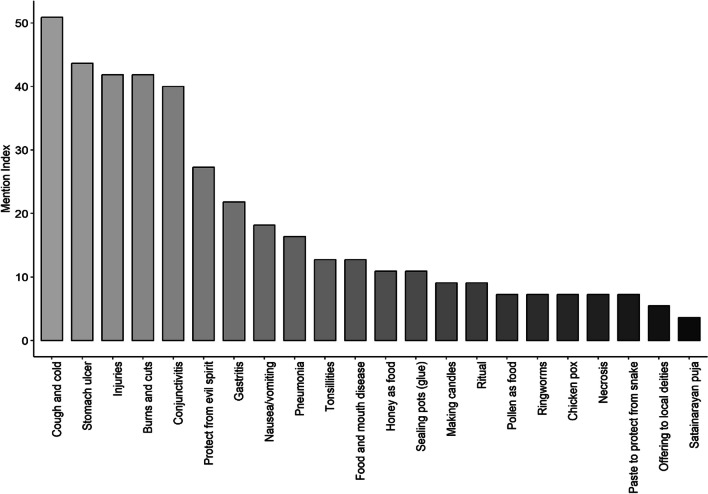
Mention index of the uses of the stingless bee and their hive products

The most mentioned uses were for medical problems such as common cough and cold, stomach ulcer, injuries and cuts and burns, and conjunctivitis and the least were for cultural purposes as shown in Fig. 4. Among the informants, 20% reported consuming stingless bee honey as the source of food and 12.7% reported using pollen as a source of protein. Furthermore, 92.7% of the participants reported using stingless bee honey to treat common coughs and colds. Whereas 80% of the participants reported using stingless bee honey to treat stomach ulcers, 76.3% reported its use to treat pains due to injuries, and 72.7% reported its use to heal from conjunctivitis. Similarly, 76.3% of the informants use it to treat skin burns and cut wounds, 12.7% of them use it to treat the ringworm infection, 23.6% of them use it for tonsillitis, and 12.7% of them use it for the treatment of the smallpox. It is also noteworthy to mention that 32.7%, 29%, and 40% of the informants use smoke from the entrance tube paste to treat nausea, pneumonia, and gastritis, respectively.

In veterinary uses, 23.6% of the informants use stingless bee honey to treat foot and mouth disease (FMD) in cattle and 12.72% for treating foot necrosis. 16.3% of our informants also use wax to prepare locally made candles, and 20% of them use it to mend holes in cooking pots. 12.7% of the total informants believe in pasting stingless bee entrance tubes on machetes to protect them from snake bites, and 49% of them practice making amulets from bee products to ward off evil spirits. Lastly, in religious contexts, 12.7% of the informants use honey in "Puran," while 9% use honey in rituals associated with offering to local deities. Moreover, 7% of them use honey in “*Satyanarayana Puja”*.

### Relative cultural importance

**Fig. 5:**
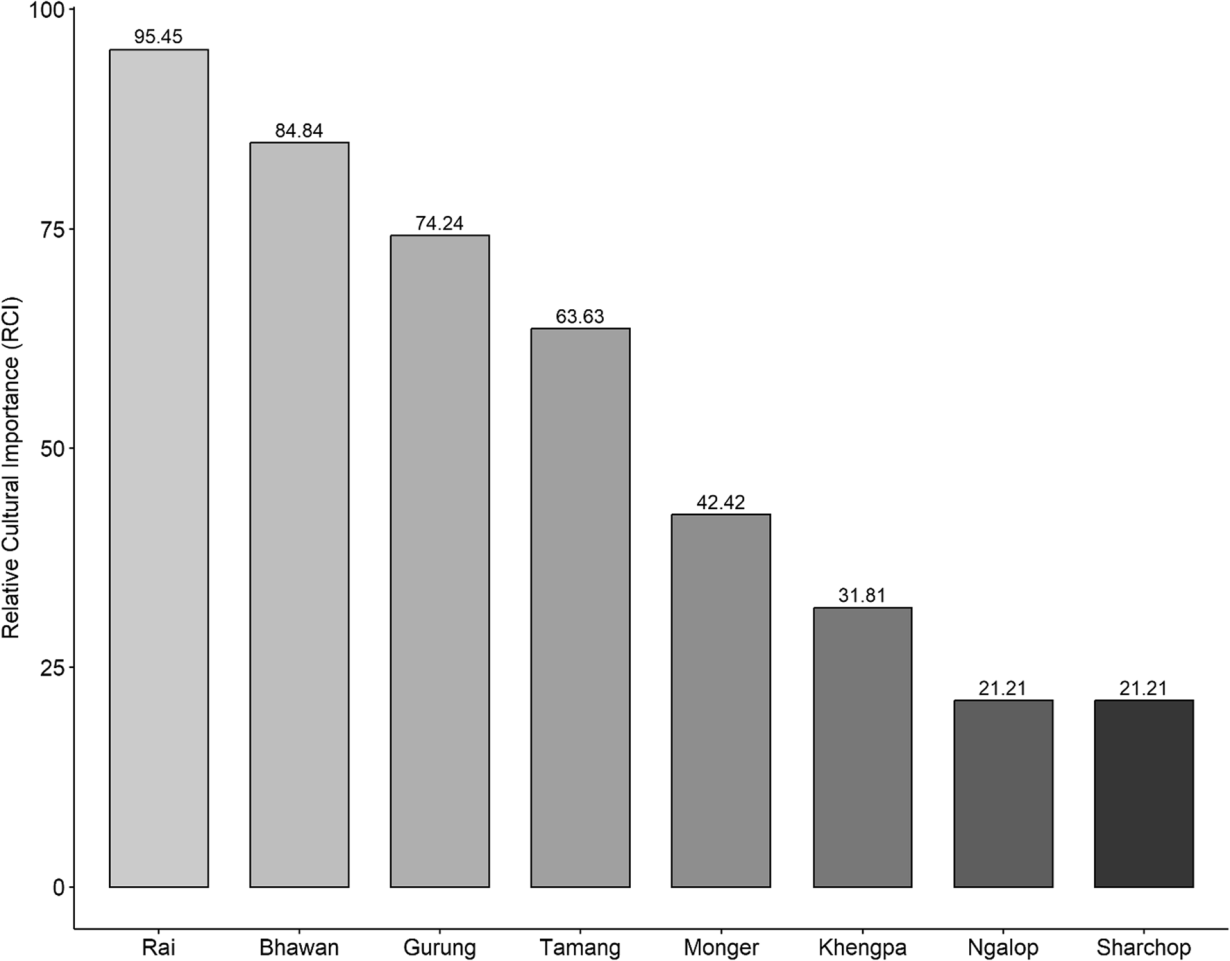
The relative cultural importance index (RCI) of stingless bees and their hive products among ethnic communities in Bhutan

The relative cultural importance index (RCI) ranged from 21.21 in the *Sharchop* and the *Ngalop* communities in east and west Bhutan to 95.45 in the *Rai* communities of southern Bhutan (Chhudzom, Jigmechoeling, Samtenling, and Logchina). The *Bhawan* and the *Gurung* people have moderate uses of stingless bees, while the *Tamang* communities have intermediate uses of stingless bees. The *Sharchops*, the *Khengpas*, and the *Ngalops* have a relatively small number of informants who use stingless bee products and the uses mentioned were less. We observed a significant difference in the relative cultural importance index among the *Bhawan*, the *Rai*, the *Gurung*, the *Tamang*, the *Mongar*, and the *Khengpas*. Dunn’s pairwise test shows a significant difference between the eight ethnic communities (*p* = 0.04) (Fig. 5).

### Domestication and sustainable use of stingless bees in Bhutan

Domestication of stingless bees is a rare activity in the western and eastern regions of Bhutan. Meanwhile, meliponiculture is more common in southern Bhutan and most of the households rear at least one or more colonies of stingless bees throughout the region. We documented sixty domesticated colonies across different areas in southern Bhutan, where participant communities have been engaged in meliponiculture for a considerable duration. The Lhotshampa community practices meliponiculture by collecting natural stingless bee nests and transferring them to their homes, where they are turned into log hives and domesticated within the boundaries of their backyard. They place a significant focus on the spiritual essence of stingless beekeeping, intertwining cultural beliefs and rituals with their practices. Additionally, they prioritize the economic dimensions of beekeeping, emphasizing its role in sustaining their livelihoods and economic well-being. The Ngalop, Sharchop, and Khengpa community, on the other hand, take a different approach, obtaining stingless bee honey and related items directly from wild colonies without disturbing their natural habitats. In essence, meliponiculture manifests as a separate practice among these ethnic groups, ranging from the Lhotshampas' domestication of stingless bees within the household setting to the other communities' reliance on honey and bee products collected from wild. *Lepidotrigona arcifera* (*Sheto/Piolo Putka*) is the most popular reared species in Chhudzom and Logchina village. This study recorded the distribution of *L. arcifera* from 300 to 1845 m. The people of Jigmechoeling gewog rear *T. gressiti*, *L. arcifera*, and other yet to be determined cryptic *Tetragonula* species. We recorded the distribution of *Tetragonula* spp. from 550 to 980 m.

Our informants observed a huge decline in the number of natural stingless bee colonies over the years. Among the informants, 25% of them collect the natural colonies from the forest and transfer them in modified box hives (Fig. 2B), while 75% of them just exploit the wild colonies through the destructive extraction practice (Fig. 2C). Due to the lack of management practices, the extraction of honey and propolis often results in the loss of the entire colony, posing a threat to the survival of the species in the microhabitat. Local people also practice collecting the natural colonies in wooden logs and keep them in their backyards without any modifications (Fig. 2D). In general, no beekeepers have practiced modern meliponiculture to propagate the colonies other than transferring wild colonies to locally modified wooden bee boxes. This study recorded the price of stingless bee honey ranges from Bhutan Ngultrum (Nu.) 7500 per 750 ml for *L. arcifera* honey and Nu.12000 per 750 ml for *T. gressiti* honey.

## Discussion

This study recorded detailed knowledge of ethno-medicinal uses of stingless bees and their hive products among the several ethnic communities of Bhutan for the first time. The ethnic communities of southern Bhutan are actively engaged in traditional meliponiculture and enriched with knowledge associated with stingless bees. As per one of our informants, “*beekeeping knowledge is transmitted either culturally or orally in Bhutan. However, an increasing number of rural to urban migration among youth and lack of promotion and conservation efforts from the government are concerning to protect and preserve stingless bees in Bhutan.”* The traditional utilization of stingless bees and their hive products, as documented here in Bhutan, offers clear evidence of the socio-cultural significance these bees hold for local communities. This is particularly evident among the Rai, Bhawan, and Gurung communities, as indicated by their elevated RCI values (Fig. 4). The indigenous knowledge regarding the use of stingless bee products within the *Lhotshampas* communities, who are of Nepalese origin, has been transmitted across generations. There is a possibility that this knowledge has disseminated to other communities, especially in regions where awareness about stingless bees exists. Athang is situated on the border of Tsirang district (not included in the study site). In Tsirang, the majority of the population comprises *Lhotshampas*, and traditional meliponiculture is practiced at the household level with one or two hives. Similarly, Zhemgang shares border with Sarpang district, and the communities of Zhemgang could have acquired the knowledge from the Lhotshampas of Sarpang. This assumption of knowledge transfer from the *Lhotshampas* is deduced since there is no cultural use of the stingless bees by the *Ngalops*, the *Sharchops*, and the *Khengpas* except for medicinal purposes. The people of eastern districts could have acquired this knowledge from either the *Lhotshampas* or the *Khengpas* through cross-cultural exchanges as there is no record of the government agencies playing any part on the management and conservation of stingless honeybees. Moreover, there is no domestication of stingless bees in the region of the three ethnicities due to their strong belief in Buddhism. From a Buddhist standpoint, harvesting and consuming honey are viewed as sinful [33], given the reverence for the diligent efforts invested by the small creatures in the process of honey production. This sentiment extends to all forms of beekeeping and is a contributing factor to the country's honey deficit. Despite the Royal Government of Bhutan introducing modern beekeeping by importing Apis mellifera in 1980 [56], the reluctance to harvest honey persists. Moreover, there is limited awareness among our farmers regarding pollination services, with 60% of farmers in the western apple-growing regions being uninformed about apple pollination and the significance of pollinators [57]. In the southern region, predominantly inhabited by Hindu communities, there exists a profound respect for honeybees due to their cultural significance. Honey holds importance in religious ceremonies such as Puran and Satyanarayana puja. Additionally, it is highly valued as a crucial ingredient in folk medicine, used to treat ailments like cough and cold, stomach ulcers, minor wounds, and asthma [58]. Unlike, some ethnic communities in India [29, 30] and Nepal [8], the ethnic communities of Bhutan have recorded more specific uses of stingless bees, indicating a heavy dependence on natural resources for their everyday life. Our studies support the fact that underprivileged and economically less stable ethnic communities (for example, the *Rai* community in Bhutan) are more dependent and experts on using and harvesting locally available natural products [59]. In fact, modern meliponiculture could be used as a tool to alleviate the economic marginalization of poor households and to promote local conservation efforts sustainably [60].

Communities in Bhutan have discovered that the use of raw honey from stingless bees aids in the healing of cuts and burns, even though they lack awareness of the specific mechanisms involved in the healing process. This healing property is attributed to the phenolic compound, antioxidant activities, and antimicrobial properties of the stingless bee honey [61]. Furthermore, it is employed in the treatment of stomach ulcers, possibly attributed to the anti-inflammatory properties inherent in this honey [62]. Additionally, livestock is a major component of livelihood for the farmers in Bhutan and stingless honeybees contribute to the treatment of Foot and Mouth Disease (FMD) and foot rot disease. This is attributed to their antimicrobial properties and the prevention of wound drying, given their moisturizing properties [63]. Stingless bee honey-based hydrogel has shown the potential to be a good wound dressing [64]. Much like the beliefs held by ethnic communities in Bhutan regarding the efficacy of stingless bee products for snake bites, the Naga tribes in Nagaland, India, also utilize propolis to treat snake and spider bites [65]. Hence, products from stingless honeybees function as first aid in rural communities. The stingless bee honey has high levels of flavonoids and polyphenols [66] as they can visit numerous floral species due to their small body size. This is one reason why the price of stingless bee honey is higher compared to *Apis mellifera* honey.

Meliponiculture is still in the infant stage in the Indian sub-continent [30], and such activity is rare and not yet documented well in Bhutan [33]. This study demonstrates that the local ethnic communities in Bhutan have extensive knowledge of ecology and the natural history of stingless bees, but there is a lack of technical support and sustainable practices as evident in other Asian countries such as China [52], Nepal [8], and India [30]. Ethno-medicinal uses reported by our informants extensively fall within similar categories as reported in other regions of the world [1, 2, 50] where they have already developed a promising meliponiculture industry. While the uses of putka honey are diverse, encompassing cultural applications, there are only a limited number of individuals practicing traditional meliponiculture in the country. This is mainly due to the lack of technical support in meliponiculture and the market availability. The One Gewog One Product (OGOP) initiative was launched under the Queens project in 2015, and it has been a purchaser of stingless bee honey to bolster products crafted with local wisdom and traditions from rural communities [56]. Additionally, Bio-Bhutan, a private enterprise, has played a significant role as one of the primary purchasers of stingless bee honey. However, the market has been adversely impacted by COVID-19, and individuals engaged in small-scale meliponiculture with 20–30 hives are facing challenges in selling honey. Therefore, government intervention is deemed essential, particularly in providing technical expertise in modern meliponiculture to enable economies of scale for production targeting the export market. The adoption of modern meliponiculture could mitigate the harmful effects of destructive wild harvesting, a primary contributor to population decline in many countries [53]. Simultaneously, it could serve as a source of income for rural farmers while offering essential pollination services. Preserving the indigenous knowledge such as the identification of stingless bees by entrance tube, nesting behavior, and recognizing the types of trees they typically inhabit, is crucial for conservation efforts.

## Conclusion

This study represents the first steps toward recognizing the importance of stingless bees in terms of ethno-medicinal uses and cultural importance among different ethnic communities in Bhutan. To develop a sustainable meliponiculture in Bhutan, we need to build on the existing indigenous knowledge to further explore the diversity and distribution of the stingless bees. We also need to utilize the rich resources from our informant’s experiences in finding the location of the nests. Accumulated knowledge from various ethnic communities about diverse nest characteristics, bee behaviors, production capacities, and management practices would prove highly valuable for initiating modern and sustainable meliponiculture in Bhutan. Recognized for its proactive conservation initiatives, underpinned by strong Buddhist beliefs and Hindu reverence for honeybees, Bhutan has the potential to be a key conservation area for stingless bees in the future. Additionally, the rich botanical biodiversity and pristine forest of Bhutan could serve as a unique source of medically and nutritionally dense stingless bee honey. While this research focused on specific areas, it is acknowledged that stingless bees are present in other parts of Bhutan, and a more comprehensive taxonomic and ecological study is needed. Therefore, further studies are essential to explore the broader landscape of Bhutan.

## Data Availability

All the data are available in this article.
