# Correction: Ethno-medicinal uses and cultural importance of stingless bees and their hive products in several ethnic communities of Bhutan

**DOI:** 10.1186/s13002-024-00694-9

**Published:** 2024-05-28

**Authors:** Thubten Gyeltshen, Chet P. Bhatta, Tulsi Gurung, Pelden Dorji, Jigme Tenzin

**Affiliations:** 1 Department of Forest Science, College of Natural Resources, Royal University of Bhutan, Punakha, Bhutan; 2 Department of Biology, Radford University Carilion, 101 Elm Avenue, Roanoke, VA USA; 3 Department of Agriculture, College of Natural Resources, Royal University of Bhutan, Punakha, Bhutan; 4 Department of Animal Science, College of Natural Resources, Royal University of Bhutan, Punakha, Bhutan

## Correction: Journal of Ethnobiology and Ethnomedicine (2024) 20:42 10.1186/s13002-023-00639-8

Following publication of the original article, the authors flagged the following two errors to the journal:

1) Abstract, Methods section

Methods

Ethnographic research was conducted to document the ethno-medicinal uses and cultural importance of stingless in Bhutan.

Should be

Methods

Ethnographic research was conducted to document the ethno-medicinal uses and cultural importance of stingless bees in Bhutan.

2) Figure 2, caption

A Domestication of stingless bee in Chhudzom Gewog. B–E. Destructive extraction of stingless bees’ hive products from wild colonies. F Wax and propolis collected for traditional use Senior author (right). G Interviewing with the Bhawan communities of Bhutan in Chhudzom Gewog

Should be

A Domestication of stingless bee in Chhudzom Gewog. B–E. Destructive extraction of stingless bees’ hive products from wild colonies. F Wax and propolis collected for traditional use. G Interviewing with the Bhawan communities of Bhutan in Chhudzom Gewog (Senior author, on the right)

These errors have since been fixed in the published article. The authors thank you for reading this erratum and apologize for any inconvenience caused.
